# Supplementary material for: Transcriptome Analysis Reveals Vimentin-Induced Disruption of Cell–Cell Associations Augments Breast Cancer Cell Migration
Source: Cells. 2022 Dec 13;11(24):4035. doi: 10.3390/cells11244035 (PMC9776984; doi:10.3390/cells11244035)
Supplement: Supplementary file 1 [file cells-11-04035-s001.zip › DEGs and enrichment analysis.zip!/DEGs and enrichment analysis/WT_VIMvsVector KEGG/DOWN/WT_VIMvsVector_DOWN.pathway_map.zip!/WT_VIMvsVector_DOWN.pathway_map/src/hsa05202.html]

hsa05202


- hsa:5468

- Down regulated genes

ENSG00000132170(-1.64958508555999)

- hsa:3486

- Down regulated genes

ENSG00000146674(-2.15078068951002)

- hsa:3576

- Down regulated genes

ENSG00000169429(-3.18264312774765)

- hsa:5468

- Down regulated genes

ENSG00000132170(-1.64958508555999)

- hsa:5468

- Down regulated genes

ENSG00000132170(-1.64958508555999)

- hsa:330

- Down regulated genes

ENSG00000023445(-2.12138419902495)

- hsa:5218

- Down regulated genes

ENSG00000058091(-2.17909865322237)

- hsa:3248

- Down regulated genes

ENSG00000164120(-3.89644724263271)

Close
